# Collagenous Colitis and Spondylarthropathy

**DOI:** 10.1155/2012/620241

**Published:** 2012-06-03

**Authors:** Kaouther Ben Abdelghani, Hana Sahli, Leila Souabni, Selma Chekili, Salwa Belhadj, Selma Kassab, Ahmed Laatar, Leith Zakraoui

**Affiliations:** Rheumatology Department, Mongi Slim Hospital, Sidi Daoued, 2046 Marsa, Tunisia

## Abstract

Collagenous colitis is a recent cause of chronic diarrhea. Cooccurrence with spondylarthropathy is rare. We describe two cases: one man and one woman of 33 and 20 years old were suffering from spondylarthropathy. They then developed collagenous colitis, 4 and 14 years after the onset of spondylarthropathy. The diagnosis was based on histological features. A sicca syndrome and vitiligo were observed with the female case. The presence of colitis leads to therapeutic problems. This association suggests a systemic kind of rheumatic disease of collagenous colitis.

## 1. Introduction

The collagenous colitis (CC) is a clinicoanatomic entity of recent individualization which was described for the first time in 1976 by Lindström [1]. In the past, CC was thought to be a rare disorder, and very little was known about its etiology or epidemiology. Many recent publications have shown that the incidence of CC is increasing [2]. A number of autoimmune diseases were reported to be associated with CC, in 17 to 40% of the cases [3]. However, spondylarthropathy (SpA) associated with CC has rarely been reported before. Here, we describe two cases of CC in two patients treated for an ankylosing spondylitis (AS).

## 2. Case Presentation

### 2.1. Case 1

Mrs KZ, aged 33, consulted in 1996 for a diffuse rachialgia associated with an inflammatory left fessalgie evolving since 10 years. There were no peripheral joint manifestations. The X plain radiography of the pelvis was normal. CT scan showed bilateral sacroiliitis. The HLA typing had found a haplotype A2*∖*B40*∖*B45. Thus, the diagnosis of SpA was established according to the ESSG criteria (European Spondylarthropathy Study Group). The patient was treated with nonsteroidal anti-inflammatory drugs (NSAID) with amendment of the pains. One year later, a chronic watery diarrhea with abdominal pains had occurred. The colonoscopy as well as the bacteriological and parasitological examinations of the stools was normal. However, the colic biopsy showed a typical aspect of CC: a thickened subepithelial collagen layer whose thickness was greater than ten microns, increased intraepithelial lymphocytes, and inflammation in the lamina propria consisting mainly of lymphocytes and plasma cells. Other associated extra-articular manifestations were also noted: an ocular sicca syndrome confirmed by the Schirmer test (normal salivary biopsy of glands) and a vitiligo of the hands and forearms. The NSAIDs were then stopped, and the patient was treated with sulfasalazine (SLZ) associated with corticosteroids (10 milligrams of equivalent prednisone/day) with resolution of the enteric symptoms and marked improvement of the joint disease, as proven by the BASDAI score which decreased from 8 to 4. In the year 2000, a symmetrical polyarthritis of the large and small joints in the lower limbs appeared. No radiological erosion had been detected and the immunological investigation (rheumatoid factor and antinuclear antibodies) remained negative. The patient did not have a concomitant diarrheic flare-up. A treatment with NSAIDs, SLZ, and a low dose of corticosteroids, and then with methotrexate (10 and 15 mg per week) were attempted but with no positive response. Because of the resistance to conventional therapies, TNF alpha inhibitor was then started. The patient received at first infliximab at a dose of 3 mg/kg then at 5 mg/kg every 8 weeks. The response was excellent, however, and at the 12th perfusion, the patient developed a toxidermia confirmed by a skin biopsy, thus motivating the switch to etanercept. After 3 months, the disease remained active (BASDAI at 8). A second switch towards adalimumab is currently underway.

### 2.2. Case 2

Mr BA, 20-year-old, was followed for a juvenile SPA (at the age of 16) with sacroiliitis and bilateral coxitis. Besides, HLA typing was not performed. In 1999, that is, four years after the beginning of his disease, the patient's disease was active under NSAID with a BASDAI at 6. At that time, the patient also developed a sudden onset of persistent diarrhea, made of watery but not bloody stools. Colonoscopy was unremarkable. Systematic colonic biopsies had revealed a thickened subepithelial collagen layer and diffuse inflammation of the lamina propria with prominent intraepithelial lymphocytic infiltration, findings consistent with CC. The NSAIDs were stopped and SLZ was started at a dose of 3 g/day, resulting in a marked clinical improvement with a BASDAI score at 3.5 after 3 months. Since then, the patient no longer had diarrhea with a current decline of 11 years.

## 3. Discussion

The CC is one of the rare causes of chronic diarrhea. Clinically, it is characterized by watery, chronic, and nonbloody diarrhea. Its diagnosis, however, remains histological and the microscopic examination of mucosal biopsies reveals characteristic histopathological changes: an abnormal thickening of the subepithelial collagen layer (>10 *μ*m), as well as a lymphocytic infiltration of the epithelium and the lamina propria [4]. The etiology of CC remains enigmatic and is multifactorial with different elements being more influential in different individuals. Recent data point to a differentiation abnormality in the colonic mucosa fibroblasts, although the exact mechanism that initiates this abnormality remains unknown. In this regard, levels of inflammatory mediators are increased in the setting of CC. These include metalloproteinases, growth factors (transforming growth factor and vascular endothelial growth factor), cytokines, nitric oxide, and prostaglandins [5]. Currently, responsibility of several drugs has been questioned, some with strong clinical and/or histological evidence suggesting causality. Thus, a paper had proposed a scoring system for drug-induced microscopic colitis, in this case CC, adapting existing criteria of drug causality and review the literature using this framework. Based on this paper, NSAID was identified with high likelihood of inducing CC [6]. This etiology had been considered in our first case. Indeed, the time course favors drug effect since diarrhea occurred right after starting NSAIDs and this treatment was then stopped. However, NSAID can themselves induce diarrhea as an adverse effect, and the coexistence of CC with NSAID usage might in fact be coincidental. Immune mechanisms seem clearly to be involved in the pathogenesis of CC. Evidence includes the finding of epithelial damage closely related to the increase of CD8 TcR alpha beta intraepithelial lymphocytes [5], and the abnormal surface membrane expression of class II major histocompatibility complex (HLA-DR) on the colonic epithelial cells [7]. A higher prevalence of antinuclear antibodies has also been found in patients with CC compared with a healthy control population [8]. The response of CC to corticosteroids also supports the hypothesis that autoimmune mechanisms may be involved. In addition, a high proportion of patients with CC have one or more autoimmune disorders, as in our cases. However, the association with a sicca syndrome can be explained by an excess of collagen. Indeed, in a prospective study of 7 cases of CC, four patients had sicca syndrome and the histological finding of the salivary glands was consistent with an excess of collagen [9]. Actually, in a retrospective study of 163 cases of CC, an associated autoimmune disease was observed in 40% of cases [10]: these particularly dealt with rheumatoid arthritis (*n* = 16), autoimmune thyroiditis (*n* = 14), celiac disease (*n* = 14) and finally diabetes mellitus (*n* = 9). In a recent large analysis of 171 case of CC, thyroid disease, celiac disease, rheumatoid arthritis, Raynaud/CREST syndrome were found to have a significantly higher occurrence in CC compared to the control group [11]. This association was also reported with the systemic lupus erythematosus [12, 13] and the systemic sclerosis [14–16]. Some cases of erosive synovitis associated with CC have been described but seem rare and non-specific. A literature search revealed other reports of CC with peripheral seronegative arthritis, at least 20 cases of oligoarthritis or polyarthritis, and 2 cases of monoarthritis [10, 17–19]. Other findings have shown a higher frequency of this arthritis, of the order of 7% [20]. Most of the authors actually consider this joint disease as an enteropathic arthritis, as observed with the other inflammatory bowel disease [21]. Nevertheless, the association of a CC with an authentic SpA remains exceptional. To our knowledge, only 11 cases who met the ESSG classification criteria for SpA were reported in the literature (Table 1) [3, 17, 18, 21–27].

The SpA, when associated with CC, is characterized by high frequency of the axial and peripheral involvement, as seen in our first case. Even if CC is more prevalent in women of about fifty, there is no female predominance [2]. The CC may precede, occur concomitantly or follow-up the disease joint [3]. In our two patients, the gastrointestinal manifestation occurred 4 and 14 years after the onset of SpA. The HLA-B27 is present in half the cases. The synovitis is usually nonerosive, nondeforming, and seronegative [3], although 2 cases of erosive arthritis have been reported (two men, aged 51 and 58, respectively, suffering from asymmetrical polyarthritis).

The joint disease usually responds to NSAIDs, SLZ, and low dose of oral glucocorticoids [3] but no well-codified treatment regimen is set. NSAIDs could induce a digestive flare in analogy with chronic inflammatory bowel diseases and raise a major therapeutic problem. Different treatments of the CC have been tried. At present, budesonide has the best-documented efficacy in treating active CC but there is a high relapse rate when medication is tapered or ceased [28]. SLZ has also been used with some positive results as seen in our two patients. Immunosuppressive agents, such as 6-mercaptopurine, azathioprine, and methotrexate are usually considered in those patients who are refractory to or dependent on steroids [5]. Recently, anti-TNF therapies were effective in CC with severe symptoms refractory to standard medical therapy [29]. To the best of our knowledge, our first case is the first report of using TNF alpha blocker in the association of CC and SpA, with effectiveness of antibodies and a failure of the soluble receptor, as is typically observed in inflammatory bowel disease.

## 4. Conclusion

The two observations highlighted above show that the CC may be associated with various autoimmune diseases. The entanglement of the CC to an authentic SpA and a vitiligo suggests autoimmune mechanisms common to these conditions. This association, albeit rare, deserves to be known because of therapeutic implications.

## Figures and Tables

**Table 1:** Characteristics of the 11 cases with CC and SpA and ours patients.

Reference	Sex	Age	Peripheral joint involvement	Axial involvement	Extra intestinal features	Erosive synovitis	HLA B27
[21]	W	33	Symmetric oligoarthritis	Sacroiliitis	Hypothyroidism sicca syndrome Raynaud	0	+
[18]	M	58	Asymmetric polyarthritis	Sacroiliitis	Psoriasis	0	−
[24]	W	51	0	Sacroiliitis	0	0	+
[25]	M	79	Symmetric oligoarthritis	Sacroiliitise	Psoriasis	0	NS
[17]	M	51	Asymmetric polyarthritis + Dactylitis + Enthesopathy	Sacroiliitis + lumbar and cervical ankylosis + atlantoaxial subluxation	Hypothyroidism Vitiligo	+	−
[22]	M	58	Asymmetric polyarthritis	Cervical ankylosis	Psoriasis	+	−
[26]	W	48	Diffuse arthralgias	Sacroiliitis	0	0	−
[27]	M	46	Symmetric oligoarthritis + Dactylitis + Enthesopathy	Sacroiliitis	0	0	+
[3]	W	37	Asymmetric oligoarthritis	Sacroiliitis + lumbar ankylosis	0	0	+
[3]	M	47	Asymmetric oligoarthritis	Sacroiliitis	0	0	−
[23]	W	53	Symmetric polyarthritis	0	Retrobulbar neuritis + Psoriasis	0	+
Our case 1	W	33	Symmetric polyarthritis	Sacroiliitis	Sicca syndrome + Vitiligo	0	−
Our case 2	M	20	0	Sacroiliitis + spine ankylosis	0	0	NS

NS: not studied; W: woman; M: man.
